# Bibliometric mapping and clustering analysis of Iranian papers on reproductive medicine in Scopus database (2010-2014)

**Published:** 2016-06

**Authors:** Soheila Bazm, Seyyed Mehdi Kalantar, Masoud Mirzaei

**Affiliations:** 1 *School of Public Health, Shahid Sadoughi University of Medical Sciences, Yazd, Iran.*; 2 *Research and Clinical Center for Infertility, Shahid Sadoughi University of Medical Sciences, Yazd, Iran.*; 3 *Yazd Cardiovascular Research Centre, Shahid Sadoughi University of Medical Sciences, Yazd, Iran*

**Keywords:** *Reproductive medicine*, *Bibliometric analysis*, *Co-authorship networks*, *Co-word analysis*

## Abstract

**Background:**

To meet the future challenges in the field of reproductive medicine in Iran, better understanding of published studies is needed. Bibliometric methods and social network analysis have been used to measure the scope and illustrate scientific output of researchers in this field.

**Objective:**

This study provides insight into the structure of the network of Iranian papers published in the field of reproductive medicine through 2010-2014.

**Materials and Methods:**

In this cross-sectional study, all relevant scientific publications were retrieved from Scopus database and were analyzed according to document type, journal of publication, hot topics, authors and institutions. The results were mapped and clustered by VosViewer software.

**Results:**

In total, 3141 papers from Iranian researchers were identified in Scopus database between 2010-2014. The numbers of publications per year have been increased from 461 in 2010 to 749 in 2014. Tehran University of Medical Sciences and "Soleimani M" are occupied the top position based on Productivity indicator. Likewise "Soleimani M" was obtained the first rank among authors according to degree centrality, betweenness centrality and collaboration criteria. In addition, among institutions, Iranian Academic Center for Education, Culture and Research (ACECR) was leader based on degree centrality, betweenness centrality and collaboration indicators.

**Conclusion:**

Publications of Iranian researchers in the field of reproductive medicine showed steadily growth during 2010-2014. It seems that in addition to quantity, Iranian authors have to promote quality of articles and collaboration. It will help them to advance their efforts.

## Introduction

Reproductive medicine deals with prevention, diagnosis and management of reproductive problems. This field has been rapidly developing following the recent advances in infertility treatment techniques (1). Due to the new techniques such as radioimmunoassay, the antiestrogen clomiphene citrate and exogenous gonadotropin hormones, fertility clinics offer comprehensive infertility treatments ranging from the initial diagnosis and treatment of infertility to advanced reproductive techniques such as IVF, donor egg, ICSI, and PGD. "Yazd Reproductive Sciences Institute" was the first clinic of infertility in Iran established in 1990 (2). There are more than 50 infertility clinics in Iran which has a unique position in Middle East region in which remarkable progresses in infertility field has provided excellence in patient care, research, and teaching through the many highly trained academic faculties who are dedicated to their work.

Based on 2025 vision, Iran has to reach a high level of progress in clinical studies and be a scientific reference country in the area of stem cell sciences and biotechnology (3). To achieve this outstanding goal, conducting highly research programs, increasing the quantity of publications and research outputs as well as optimizing citations to Iranian papers and improvement the quality of papers is essential (3). Bibliometrics is a set of mathematical and statistical methods that offers several opportunities to support research (4). Bibliometric mapping provides a means to visualize academic output as publication and citation information for parameters of a particular field. It allows for the representation of information in ways which make relationships more obvious and easier to understand and can lead to new insight and discovery (5). Moreover cluster analysis revolves a number of different algorithms aiming to detect natural division of networks into groups (clusters), on the basis of similarity and minimize inter cluster similarity (3).

To our best knowledge, there is a lack of data concerning the bibliometric mapping and clustering analysis of research in the field of reproductive medicine in Iran. The main objective was to provide detailed map and clustering of data set related to Iranian research in the field of reproductive medicine during 2010-2014.

## Materials and methods

In this cross-sectional study, all Iranian research papers in the field of reproductive medicine indexed in Scopus database through 2010-2014 were evaluated with bibliometrics methods and Social network analysis (SNA). To have exclusive findings we used Mesh heading to find key words and synonyms of terms in this field. The search strategy of this research is presented in Appendix A. After retrieving data, at first, bibliometric methods was applied to find distribution of the publications among years, type of documents, name of journals, authors, and institutions.

The measurements of bibliometric analysis (e.g. authors, institutions, and journals) were converted to rank order using the standard competition ranking. Standard competition ranking, is a ranking system in which the mathematical values that are equal are given equal rank and the next, lesser value is given the next highest rank. In this study, only the top 10 ranked were taken into consideration. If the measurements of bibliometric analysis have the same ranking number, then a gap is left in the following ranking numbers (7).

The common performance indicator, journal impact factor (IF) and SCImago Journal Rank (SJR) were considered for top 10 ranked journals using data from the most recent years available. The journal IF was evaluated using the Journal Citation Report (JCR; Web of Knowledge) 2014 science edition by Thomson Reuters (New York, NY, USA). IF is a measure reflecting the average number of citations to articles published in science and social science journals, while SJR is a measure of scientific influence of scholarly journals that is calculated based on the number of citations received by a journal and the importance of the journals where such citations come from (8).

At the second stage, SNA was used for data analysis. SNA is a set of theories, tools, and processes for understanding the relationships and structures of a network (9). The “nodes” of a network are the people and the “links” are the relationships between people. Nodes are also used to represent events, ideas, objects, or other things (9). To answer the questions of this study, by using Vos Viewer software, mapping and clustering techniques are often used in a combined fashion, in which nodes represents individual (10). Each node is coloring based on the cluster it belongs. VosViewer is a free software (downloaded from http://vosviewer.com) that is useful for displaying large bibliometric maps in Label view, Density view, Cluster view and Cluster density view (10).

## Results

Totally, we found 3141 Iranian-based papers in the field of reproductive medicine indexed in Scopus through 2010-2014. These papers categorized in over ten different document types. There were 2810 (89.46%) articles, 190 (6.04%) reviews, and 141 (4.48%) other documents types including letters, conference papers, articles in press, notes, editorials, short surveys, book chapters, and erratum. 

Output publication of this study was published in 164 different journals. Table I displays, the top10 journals that published Iranian papers on the field of reproductive medicine 2010-2014. These top 10 journals had published 96.56% of papers.

**Bibliometric analysis**

**Figure 1:**
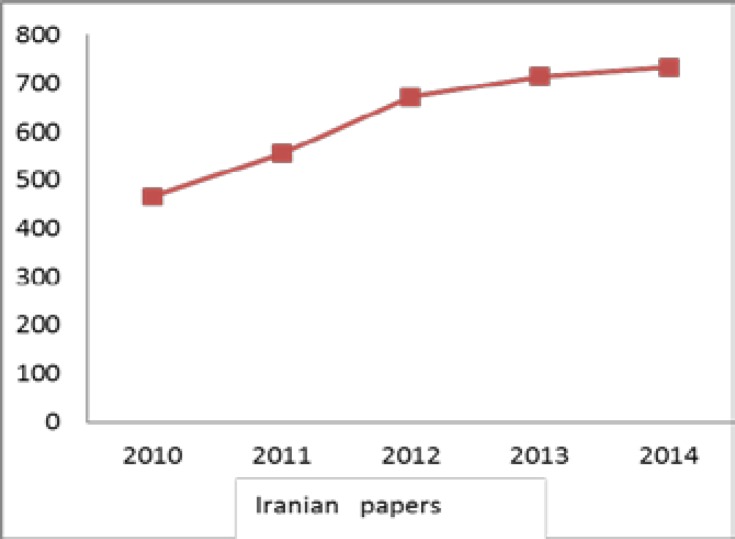
Trend of publication of Iranian researchers in the field of reproductive medicine through 2010-2014

Figure 1, presents trends in reproductive medicine researches conducted by Iranian researchers. It shows that the number of scientific papers increased steadily from 2010 to 2014, in which the year of 2014 was the peak of publication outputs. The growth pattern of literature represents slow development of publications from 2013 to 2014.

In terms of institutional productivity, Tehran University of Medical Sciences authored the most articles (855), Tarbiat Modares University along with Iranian Academic Center for Education, Culture and Research (ACER) ranked second (362) and Shahid Beheshti University of Medical Sciences ranked third (267) (Table II). In regard to author's productivity, "Soleimani M", "Baharvand H" and "Ghavamzadeh A" are three top Iranian authors that published the most number of articles.

**Social network analysis**

**Co-word network:**

To find the most popular research topics, the distribution of authors' keywords was investigated. Co-word analysis is a content analysis method that is effective way in mapping the strength of association between keywords in textual data, keywords that are located close to each other in the map often occur together, while keywords that are located far from each other, do not or almost not come together (3). In general, terms in the center of the map co-occur with many different terms and are therefore related to different topics. In contrast, terms at the edges of the map tend to co-occur only with a small number of other terms and often belong to relatively isolated field (12). 

**Figure 2:**
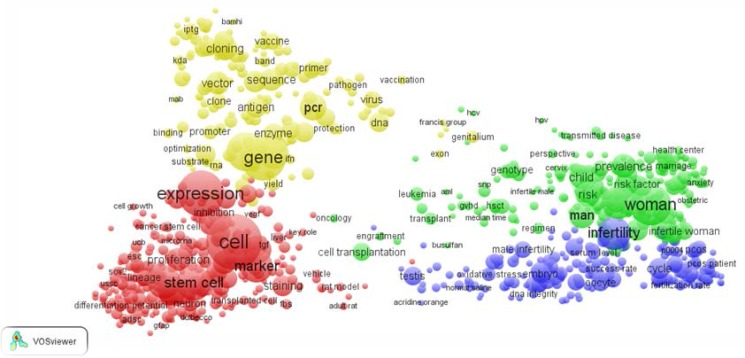
Co-word map of hot topics in the papers published by Iranian researchers in the field of reproductive medicine 2010-2014

The color and the size of a term indicate the cluster and the frequency with which the terms have been appeared respectively, the cluster size in the map influenced by many factors like the number of terms in the cluster and the frequency of occurrence of the terms (12). Total number of these keywords was 910 that encompass 4 main clusters in four colors red (public and important keywords), green, blue, and yellow (Figure 2). Most of hot topics were keywords in clusters 1 and some of them in cluster 2, 3, and 4. 

**Figure 3:**
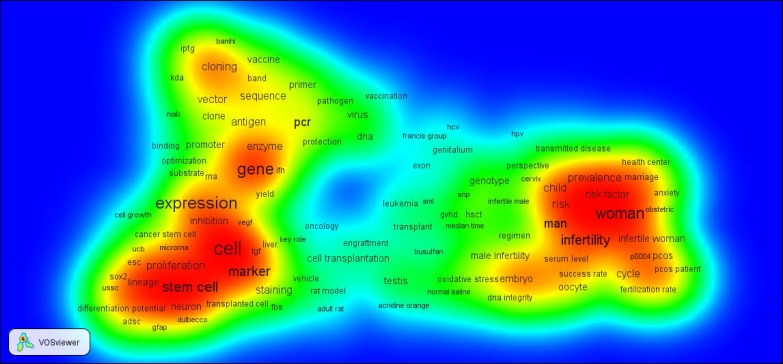
Density map of hot topics in the papers published by Iranian researchers in the field of reproductive medicine 2010-2014

According to the findings of this research subjects and keywords included cell, cloning, woman, infertility, expression, gene marker, man, and stem cell that are found in red and orange area, are hot topics. The size of each term indicates the importance of these key words in Iranian reproductive medicine researches. By moving from red area to blue in density map, density is decreased, in the other words key terms are located in red and orange area (Figure 3). Two clusters of gen and cell have locations close to each other. Likewise the other clusters in green and blue colors are close to each other too. In green cluster, woman has been connected with other terms like child, risk and risk factor that is expectable.

**Co-authorship networks**

Co-authorship network is a social network in which the authors or organizations through participation in one or more publication through an indirect path have linked to each other; the strength of relations between publications makes similarity measures and thus the possibility of making cluster. 

To analyze co-authorship networks, in this study, two criteria of centrality and betweenness centrality were measured. Centrality is one of the most important scales and frequently is used in social network analysis. Centrality measures how central the author or organization is to the network, which presents a useful view for assessing researcher's performance according to their function and roles in the network (13). Betweenness centrality is an indicator of a node's centrality in a network. It is equal to the number of shortest paths from all vertices to all others that pass through the nodes. It measures the number of times a node acts as a bridge along the shortest path between two other nodes (13).

**Co-authorship network of institutions**

Regarding the co-authorship of academic institutions in this field, the minimum articles published by each institution considered five articles. According to these criteria from the total number of 7593 institutions, 139 organizations meet the eligibility criteria and threshold; next 15 organizations without co-authorship were omitted from analyzing. Finally, 112 organizations were included in this analysis. 

The co-authorship map of Iranian institutions includes 13 clusters in different colors. Each node is labeled with the name of institutions as well as each line represented co-authorship between them. By moving from red area to blue area, density has been decreased. In the other words institutions with the high degree of co-authorship are presented in the red and orange area. Therefore institutions in these two areas have high density and are top ranked institutions based on co-authorship criteria (Figure 3). 

Degree centrality and betweenness centrality were also calculated for institutions co-authorship network (Table V). With respect to degree centrality and betweenness centrality indicators, the top institutions are identified (Table V).

Organizations with the highest degree centrality are more central in network structure and have a greater capacity to influence other institutions (12). The betweenness centrality scores represent the degree to which the node under study can function as a point of control in the communication, if a node with a high level of between was to be omitted from a network, the structure of network would be changed into otherwise coherent clusters (12).

**Co-authorship network of author network**

To draw the co-authorship network of authors in VosViewer, the minimum number of published articles by authors has been considered five. Therefore from the total number of 8758 authors, 477 of them have meet this criteria, in the next step eight authors without any co-authorship were excluded from analyzing. Therefore 469 authors remained in this analysis. Cluster analysis of researchers' co-authorship network indicates that this network includes 23 clusters in different colors. 

**Figure 4:**
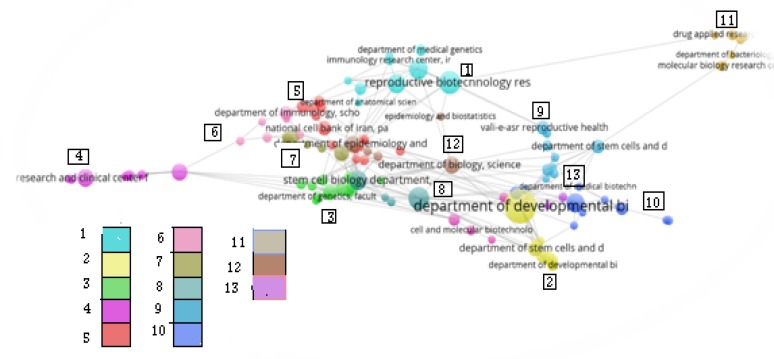
Label view of Co- authorship of Iranian institutions in the field of reproductive medicine in the period of 2010-2014

The main and important clusters in the co-authorship network of authors are appearing in bright green color by presence of "Soleimani M"; bright blue color by presence of "Baharvand H"; bright yellow color by presence of "Ghavamzadeh A" and "Alimoghaddam K" and finally bright purple color by presence of "Nasr-Esfahani MH" (Figure 4). In regard to density view, researchers with more scientific relationship are located closer to each other. 

The density of each researcher is determined based on the quantity of scientific production of them and the number as well as the importance of neighborhood's nodes. Moreover, based on the position of each node on the map the importance of it is determined. Two indicators of degree centrality and betweenness centrality were calculated for authors' co-authorship network (Table VI). Degree centrality measures how central the author is to the network as well as the role and performance of researcher tend to the other researchers in the network (13). 

Authors with higher degree centrality are more central in network structure and tend to have greater capacity to influence others. The results showed that most of authors have very low degree centrality."Soleimani, M" is the most active and visible author with the highest degree of collaboration. In regard to betweenness centrality scores, the most influential author is "Soleimani, M" again. Authors with high degree of centrality play a great role to connect the clusters of network.

**Table I.** Top 10 most productive journals published Iranian papers on reproductive medicine 2010-2014

**No.**	**Journals**	**Article (n)**	**IF** *	**SJR** ٭٭
1	Iranian Journal of Reproductive Medicine	102	-	0.188
2	International Journal of Fertility and Sterility	95	-	0.217
3	Cell Journal	57	0.233	0.187
4	Iranian Red Crescent Medical Journal	48	-	0.174
5	Iranian Journal of Basic Medical Sciences	45	1.228	0.224
6	Journal of Assisted Reproduction and Genetics	43	-	1.072
7	Journal of Reproduction and Infertility	36	0.172	0.172
8	Iranian Journal of Biotechnology	36	0.375	0.230
9	International Journal of Hematology Oncology and Stem Cell Research	35	0.133	0.133
10	Andrologia	35	-	0.458

* IF: Impact Factor

** SJR: SCImago Journal Rank

**Table II:** Top 10 most productive Iranian institutions in the field of reproductive medicine, 2010-2014

**No**.	**Institutions**	**No. of article**
1	Tehran University of Medical Sciences	855
2	Tarbiat Modares University	362
2	Iranian Academic Center for Education, Culture and Research(ACER)	362
3	Shahid Beheshti University of Medical Sciences	267
4	Daneshgahe Azad Eslami	256
5	University of Tehran	195
6	Shiraz University of Medical Sciences	194
7	Isfahan University of Medical Sciences	155
8	Tabriz University of Medical Sciences	135
9	Pasteur Institute of Iran	128
10	Shahid Sadoughi University of Medical Sciences and Health Services	124

**Table III:** Top 10 most productive Iranian authors in the field of reproductive medicine 2010-2014

**No.**	**Authors**	**No. of article**
1	Soleimani, M.	123
2	Baharvand, H	118
3	Ghavamzadeh, A.	77
4	Gourabi, H.	45
5	Nasr-Esfahani, M.H.	44
6	Alimoghaddam, K.	39
7	Zarnani, A.H.	38
8	Akhondi, M.M	36
9	Jeddi-Tehrani, M.	34
10	Salekdeh, G.H.	32

**Table IV:** View of Co-authorship of Iranian institutions in the field of reproductive medicine in the period of 2010-2014

1	Department of Medical Genetics, Tehran University of Medical Sciences, Tehran, Iran
	National Cell Bank of Iran, Pasteur Institute of Iran, Tehran, Iran
2	Department of Genetics, Faculty of Biological Sciences, Tarbiat Modares University, Tehran, Iran
3	Department of Medical Biotechnology, National Institute of Genetic Engineering and Biotechnology, Tehran, Iran
4	Department of Developmental Biology, University of Science and Culture, ACECR, Tehran, Iran
	Department of Stem Cells and Developmental Biology, Cell, Royan Institute for Stem Cell Biology and Technology, Tehran, Iran
5	Research and Clinical Center for Infertility, Shahid Sadoughi University of Medical Sciences, Yazd, Iran
6	Department of Anatomical Sciences, School of Medicine, Tehran University of Medical Sciences, Tehran, Iran
	Immunology Research Center, Iran University of Medical Sciences, Tehran, Iran
	Reproductive Biotechnology Research Center, Avicenna Research Institute, ACECR, Tehran, Iran
7	Department of Stem Cells and Developmental Biology, Royan Institute for Stem Cell Biology and Technology, ACECR, Tehran, Iran
	Vali-e-Asr Reproductive Health Research Center, Tehran University of Medical Sciences, Tehran, Iran
8	Department of Bacteriology, Tarbiat Modares University, Tehran, Iran
	Drug Applied Research Center, Tabriz University of Medical Sciences, Tabriz, Iran
	Molecular Biology Research Center, Baqiyatallah University of Medical Sciences, Tehran, Iran
9	Department of Immunology, School of Medical Sciences, Tarbiat Modares University, Tehran, Iran
10	Department of Epidemiology and Biostatistics, School of Public Health, Tehran University of Medical Sciences, Tehran, Iran
	Epidemiology and biostatistics department, Tehran university of medical sciences, Tehran, Iran
11	Stem Cell Biology Department, Stem Cell Technology Research Center, Tehran, Iran
12	Department of Biology, School of Sciences, University of Isfahan, Isfahan, Iran
13	Hematology-Oncology and Stem Cell Transplantation Research Center, Tehran University of Medical Sciences, Tehran, Iran

**Table V:** Top 10 Iranian institutions based on Centrality and Collaboration indicators

**Degree Centrality**		**Betweenness Centrality**		**Collaboration**	
**Organization**	**Freq**	**Organization**	**Freq.**	**Organization**	**Freq.**
Department of Developmental Biology, University of Science and Culture, ACECR, Tehran, Iran	60	Department of Developmental Biology, University of Science and Culture, ACECR, Tehran, Iran	1.76	Department of Developmental Biology, University of Science and Culture, ACECR, Tehran, Iran	49
Department of Biotechnology, College of Science, University of Tehran, Tehran, Iran	27	Department of Biotechnology, College of Science, University of Tehran, Tehran, Iran	0.98	Department of Biotechnology, College of Science, University of Tehran, Tehran, Iran	34
Reproductive Biotechnology Research Center, Avicenna Research Institute, ACECR, Tehran, Iran	26	Reproductive Biotechnology Research Center, Avicenna Research Institute, ACECR, Tehran, Iran	0.92	Reproductive Biotechnology Research Center, Avicenna Research Institute, ACECR, Tehran, Iran	24
Monoclonal Antibody Research Center, Avicenna Research Institute, ACECR, Tehran, Iran	22	Monoclonal Antibody Research Center, Avicenna Research Institute, ACECR, Tehran, Iran	0.97	Monoclonal Antibody Research Center, Avicenna Research Institute, ACECR, Tehran, Iran	22
Nano biotechnology Research Center, Avicenna Research Institute, ACECR, Tehran, Iran	22	Nano biotechnology Research Center, Avicenna Research Institute, ACECR, Tehran, Iran	0.96	Nano biotechnology Research Center, Avicenna Research Institute, ACECR, Tehran, Iran	21
Stem Cell Biology Department, Stem Cell Technology Research Center, Tehran, Iran	20	Stem Cell Biology Department, Stem Cell Technology Research Center, Tehran, Iran	0.89	Stem Cell Biology Department, Stem Cell Technology Research Center, Tehran, Iran	18
Isfahan Fertility and Infertility Center, Isfahan, Iran	18	Isfahan Fertility and Infertility Center, Isfahan, Iran	0.74	Isfahan Fertility and Infertility Center, Isfahan, Iran	16
Department of Molecular cell Biology, Stem Cell Technology Research Center, Tehran, Iran	13	Department of Molecular cell Biology, Stem Cell Technology Research Center, Tehran, Iran	0.78	Department of Molecular cell Biology, Stem Cell Technology Research Center, Tehran, Iran	15
Department of Hematology, Faculty of Medical Sciences, Tarbiat Modares University, Tehran, Iran	13	Department of Hematology, Faculty of Medical Sciences, Tarbiat Modares University, Tehran, Iran	0.78	Department of Hematology, Faculty of Medical Sciences, Tarbiat Modares University, Tehran, Iran	14
Department of Stem Cells and Developmental Biology, Royan Institute for Stem Cell Biology and Technology, Tehran, Iran	12	Department of Stem Cells and Developmental Biology, Royan Institute for Stem Cell Biology and Technology, Tehran, Iran	0.75	Department of Stem Cells and Developmental Biology, Royan Institute for Stem Cell Biology and Technology, Tehran, Iran	13

**Table VI.** Top 10 Iranian authors based on Centrality and Collaboration indicators

**Degree Centrality**		**Betweenness Centrality**		**Collaborations**	
**Author**	**Freq**	**Author**	**Freq.**	**Author**	**Freq.**
Soleimani M.	116	Soleimani M.	2.524	Soleimani M.	43
Baharvand H.	97	Baharvand H.	1.967	Baharvand H.	41
Ghavamzadeh A.	72	Ghavamzadeh A.	1.637	Ghavamzadeh A.	38
Alimoghaddam K.	51	Alimoghaddam K.	0.968	Alimoghaddam K.	30
Nasr-Esfahani MH.	49	Nasr-Esfahani MH.	0.827	Nasr-Esfahani MH.	29
Ghourabi H.	39	Ghourabi H.	0.536	Ghourabi H.	23
Salekdeh GH.	28	Salekdeh G.H.	0.502	Salekdeh G.H.	20
Hamidieh AA.	28	Hamidieh AA.	0.502	Hamidieh AA.	20
Jeddi-Tehrani M.	27	Jeddi-Tehrani M.	0.365	Jeddi-Tehrani M.	20
Aghdami N.	26	Aghdami N.	0.364	Aghdami N.	19

**Figure 5:**
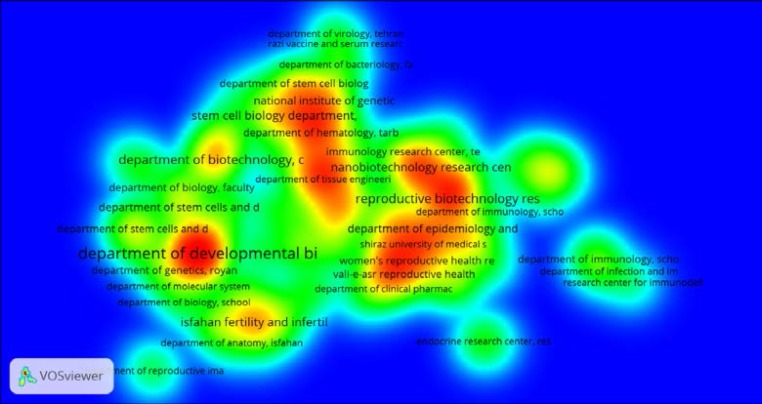
Density map of Co- authorship of Iranian institutions in the field of reproductive medicine in the period of 2010-2014

**Figure 6:**
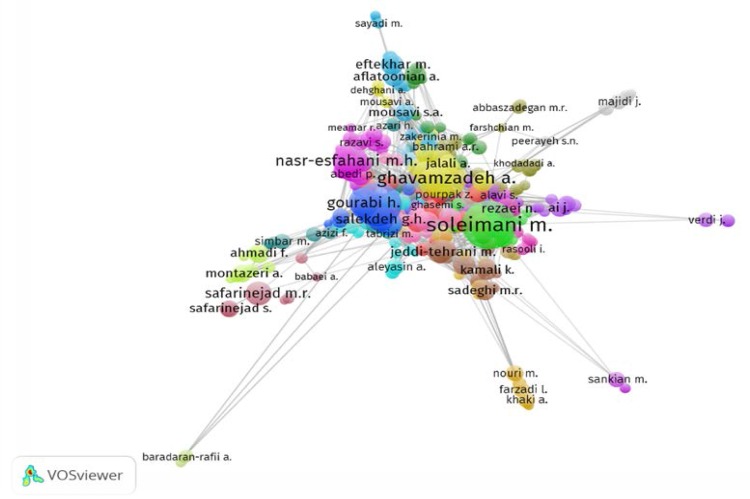
Label map of co-authorship of Iranian authors in the field of reproductive medicine, 2010-2014

**Figure 7:**
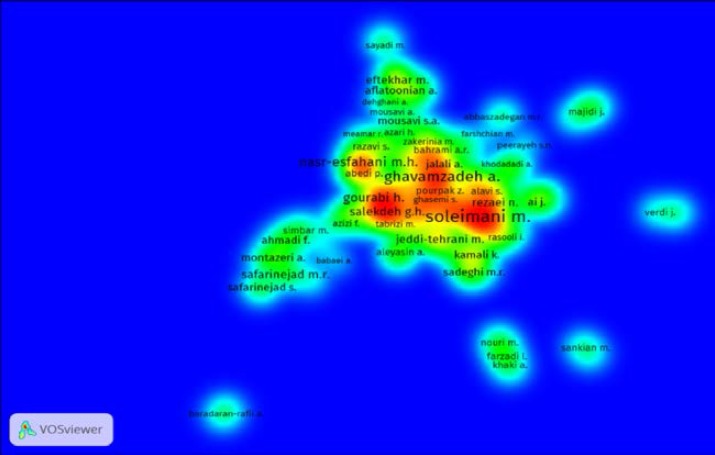
Co-authorship of Iranian authors in the field of reproductive medicine, 2010-2014

## Discussion

In this study, we have provided a supplemental evaluation of the status of reproductive medicine in Iran through 2010-2014. Such a study will lead to better understand the current and future status of reproductive medicines' research in Iran. In addition researchers can use the findings to compare the status of Iran with other countries in this regard. The total number of reproductive medicine documents retrieved and analyzed using the methodology stated was 3141. Scopus is considered as a trustful and powerful search engine with citation analysis (14). However, not all journals are indexed in Scopus and therefore publications pertaining to reproductive medicine, in these un-indexed journals were not counted.

Our analysis confirms the upward increase in research output in this field among Iranian researchers during 2010-2014. In total, there were 3141 research articles published in 164 journals. Two journals of Iranian Journal of Reproductive Medicine (IJRM) and International Journal of Fertility and Sterility hold the most number of articles in this field. Both of these two journals are outside of Science Citation Index. It seems that efforts should be made to include these Iranian journals in the ISI Science Citation Index to increase their visibility worldwide.

The co-word cluster mapping using VOS viewer visualization allowed this to be investigated by mining the title and abstract of papers. The findings suggest that for the period of 2010-2014, the studies of Iranian researchers, is structured around four important descriptive terms include cell, woman, infertility and gene keywords that are expectable. It is proved the author's keywords analysis, to be important in monitoring the development of science (3).

Our results highlighted that, among the institutions ACECR and among the authors "Soleimani M" have the most influency in this field. ACECR and its faculty members have a significant role in publishing scientific papers in the field of reproductive medicine and obtained the highest score in two indicators of centrality and collaboration. ACECR is an Iranian public non-governmental institution, established in 1980, with the mission of presenting native models for production of knowledge and technology. It includes 129 Research Groups and 113 Technical Centers (15). Reproductive Biomedicine Stem Cell Biology and Technology (Royan Institute) and Department of Developmental Biology are two of these research centers.

Centrality, productivity and collaboration of a university are mostly related to the authors who are affiliated with that university (15). In other word, institutional centrality within collaboration network emerges and develops as authors affiliated with that institutions create co-authorship links. For example "Soleimani M" is a faculty member in Department of Developmental Biology and plays a vital role in increasing the centrality of it.

The results show that the most productive institutions are mostly due to a large number of co-authored papers between productive individuals in those institutions (16). Another possible explanation for such strong links between institutions is that most authors have published their articles under different affiliations, which can increase the potential of co-authoring of those institutions. For example "Soleimani M" published his papers under the affiliation of both institutions of Department of Developmental Biology and Department of Biotechnology which can be effective in strengthening co-authoring link between these two institutions. It is noted that the strongest partnerships in the network exist between departments and institutions are located in Tehran. This finding is somewhat consistent with that of Katz who found that geographical proximity results in more collaboration (17).

Although in this study Tehran University of Medical Sciences obtained the first rank based on productivity indicator, it seems that researchers in different institutions of ACECR use different affiliation in which it was found two affiliations of ACECR and Royan Institute separately when data was retrieved in Scopus database. Based on the findings of this research, policy makers could understand the status and positions of their countries or institutions. Some studies indicated that in Iran, more than one million couples suffer from infertility. Different studies represented different prevalence of it, the latest one revealed that the rate of infertility is 13.2% which is higher than the world average (18-23). It seems that Iranian researchers should heavily participate and cooperate in research in this field in order to combat this problem. Therefore the results of this study could spell out suggestions for the reproductive medicine or reform directions. For example researchers could start their studies by focusing on hot topics in this field. In addition the findings of this study enable them to select cooperational institutions and authors.

To the best of our knowledge, this study is the first of its kind to obtain initial data regarding the publication and citation productivity in the reproductive medicine field using Scopus database; a database that is being used to evaluate the performance of institutes and their members. This study represented the current situation in this field and did not describe the features of journals, institutions or authors and do not compare the situation of Iran with other countries. 

Moreover, to attain comprehensive perspective of reproductive medicine's research in Iran, it is essential that in future bibliometric studies, evaluate other local scientific materials such as monographs, books, and dissertation theses as well as articles published in journals are not indexed in Scopus database.

## Conclusion

Our study demonstrates evidence that research productivity related to Iranian researchers in the field of reproductive medicine has increased during 2010-2014. This progress is in line with the upward scientific level in this category in the world. Due to the advantages of scientific collaboration for scientists and patients, it is essential that Iranian researchers extend national and international collaboration to promote quality of scientific outputs.
